# Resolution of tension pseudomeningocele complicating foramen magnum decompression for Chiari I malformation after ventriculoperitoneal shunt: A case report

**DOI:** 10.1016/j.ijscr.2025.111510

**Published:** 2025-06-20

**Authors:** Yi-Chen Lee, Yu-Feng Su, Hui-Yuan Su

**Affiliations:** a Division of Neurosurgery, Department of Surgery, Kaohsiung Medical University Hospital, Kaohsiung, Taiwan; b Graduate Institute of Clinical Medicine, College of Medicine, Kaohsiung Medical University, Kaohsiung, Taiwan; c Graduate Institute of Medicine, College of Medicine, Kaohsiung Medical University, Kaohsiung, Taiwan

**Keywords:** Chiari I malformation, Foramen magnum decompression, Hydrocephalus, Pseudomeningocele, Ventriculo-peritoneal shunt

## Abstract

**Introduction:**

Tension pseudomeningocele is a rare complication that arose after foramen magnum decompression in a patient with Chiari I malformation and syringomyelia. This article presents a case that was effectively managed through cerebrospinal fluid diversion methods, highlighting the mechanisms involved and the treatment option used.

**Case report:**

A 15-year-old girl came to our clinic with allodynia in the right anterior chest wall that had lasted for six months. An MRI showed a type 1 Chiari malformation along with syringomyelia from C2 to C6. After performing foramen magnum decompression and duraplasty, there was an improvement in the syringomyelia; however, the patient developed a tension pseudomeningocele in the posterior fossa. She then had a ventriculoperitoneal shunt placed, which successfully resolved the tension pseudomeningocele and the cerebrospinal fluid leakage.

**Discussion:**

Pseudomeningocele is a significant postoperative complication that can occur following foramen magnum decompression, potentially worsening neurological outcomes through mechanisms such as cord edema and the progression of syringomyelia. This condition may arise from a sustained craniospinal pressure gradient at the foramen magnum, which promotes the accumulation of cerebrospinal fluid within the pseudomeningocele cavity. As a result, cerebrospinal fluid diversion techniques, such as pseudomeningocele-peritoneal shunts or ventriculoperitoneal shunts, may be employed to reduce pressure within the cavity and restore balance to the craniospinal pressure gradient, thereby enhancing the patient's clinical status.

**Conclusion:**

The implementation of cerebrospinal fluid diversion procedures may be pivotal in addressing tension pseudomeningocele in patients with Chiari I malformation.

## Highlights

• Tension pseudomeningocele represents a potential complication following foramen magnum decompression with duraplasty in patients with Chiari I malformation.
• The underlying mechanism may be associated with a sustained craniospinal pressure gradient between the obex and the surrounding cavity.
• Resolution of the pseudomeningocele can be achieved through shunting surgery, as this procedure effectively addresses the pressure gradient.

## 1 Introduction

Chiari I malformation is defined by the herniation of the cerebellar tonsils and a disruption in the dynamics of cerebrospinal fluid at the foramen magnum [1]. The prevalence of Chiari I malformation is estimated to range from 3 to 8 cases per 100,000 individuals; notably, approximately 65 % of those diagnosed with this condition also present with syringomyelia [2]. Presently, surgical intervention, specifically foramen magnum decompression with or without duraplasty, is the preferred treatment for Chiari I malformation accompanied by syringomyelia [3]. Potential complications arising from foramen magnum decompression surgery include infection, subdural collections, epidural fluid accumulation, pseudomeningocele, hydrocephalus, and persistent syringomyelia [3,4]. The occurrence of tension pseudomeningocele following foramen magnum decompression is considered rare [1]. This report presents an exceptionally rare case of tension pseudomeningocele subsequent to foramen magnum decompression in a patient with Chiari I malformation and syringomyelia. Also, it has been developed in accordance with the SCARE Guidelines, which are designed to promote thoroughness and transparency in the reporting of surgical case studies [5].

## 2 Case report

**Fig. 1:**
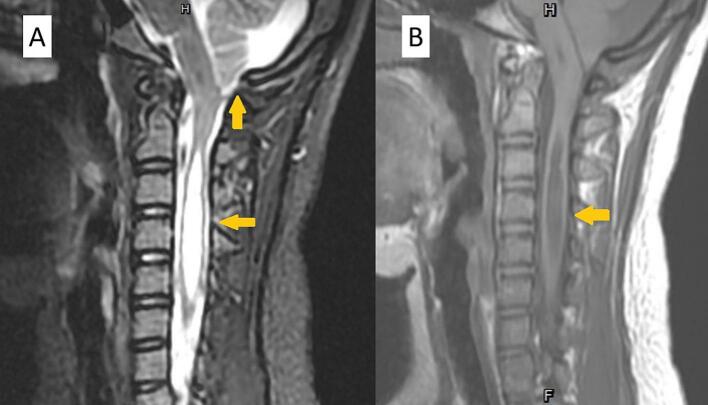
A. A sagittal T2-weighted magnetic resonance imaging (MRI) scan of the cervicothoracic region reveals a hyperintense signal within the spinal cord at the C2 to C6 levels, accompanied by observable spinal cord enlargement. B. A sagittal T1-weighted MRI of the cervicothoracic region demonstrates a hypointense signal at the C2 to C6 spinal cord levels, as well as spinal cord enlargement. Additionally, herniation of the cerebellar tonsils through the foramen magnum is observed.

**Fig. 2:**
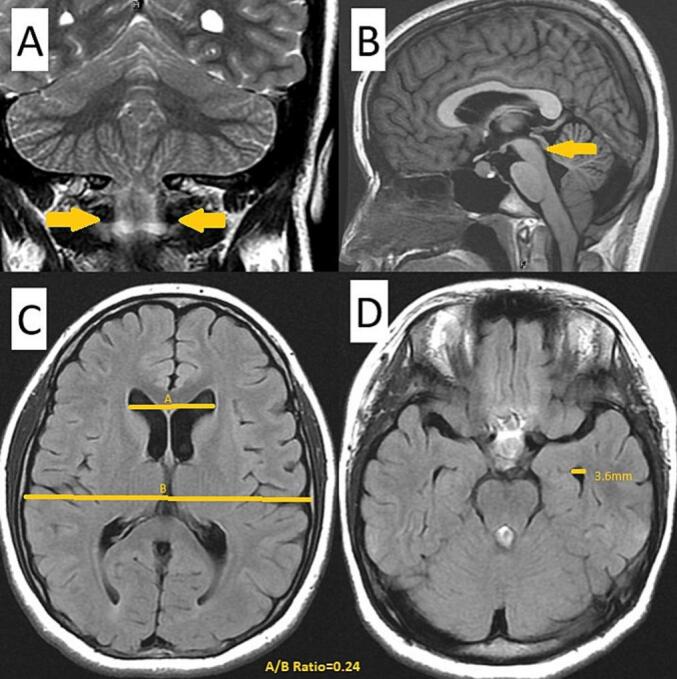
presents the results of a brain magnetic resonance imaging (MRI) examination, which demonstrates the presence of bilateral cerebellar tonsil herniation (illustrated in Fig. 2A), the absence of aqueductal stenosis (depicted in Fig. 2B), and evidence of occult hydrocephalus, characterized by an Evan's index of 0.24 (shown in Fig. 2C) and a temporal horn width of 3.6 mm (as indicated in Fig. 2D).

**Fig. 3:**
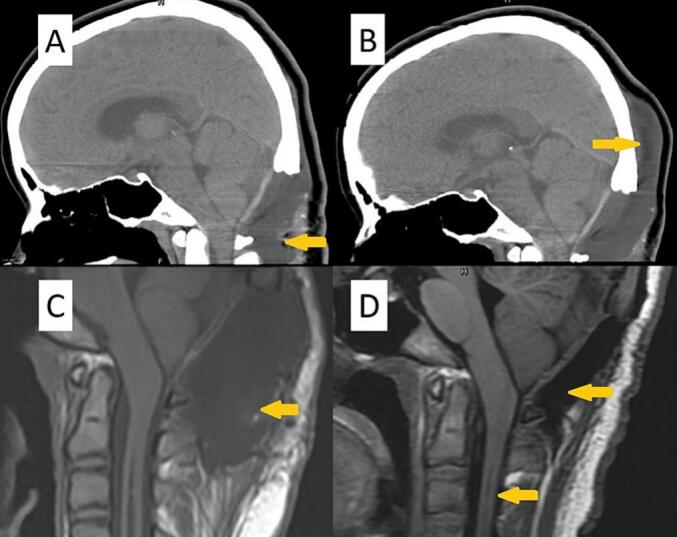
A. Three weeks postoperatively, a non-enhanced brain computed tomography scan reveals a skull defect resulting from prior foramen magnum decompression surgery, as well as a pseudomeningocele located in the posterior fossa adjacent to the duraplasty site. B. A subsequent brain computed tomography scan demonstrates an exacerbation of the pseudomeningocele, characterized by upward and epicranial extension following two weeks of conservative management. C. A T1-weighted magnetic resonance imaging of the cervicothoracic region illustrates a tension pseudomeningocele extending into the occipital subgaleal space, alongside a partial resolution of syringomyelia. D. One week subsequent to the placement of a ventriculoperitoneal shunt, there is a notable improvement in the pseudomeningocele, with sustained resolution of the syringomyelia.

**Fig. 4:**
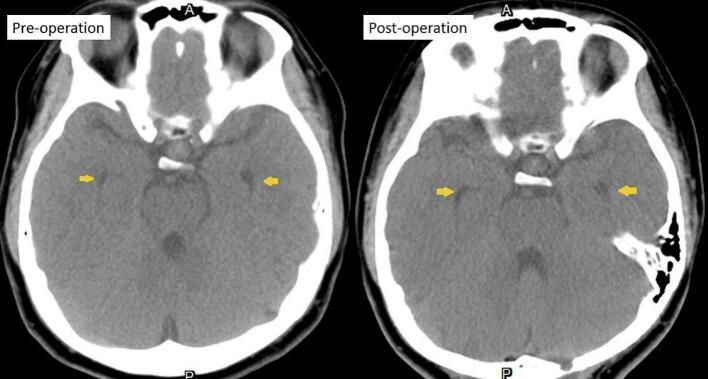
indicates that there is no statistically significant difference in the dimensions of the bilateral ventricular temporal horns when comparing preoperative and postoperative brain computed tomography images.

A 15-year-old female patient with a prior diagnosis of scoliosis presented with a tingling sensation localized to the anterior chest wall, persisting for a duration of six months. This discomfort intensified when the patient maintained a neck flexion posture for periods exceeding 15 min. Additionally, she reported episodes of chest tightness and soreness in the posterior neck and bilateral shoulders. Upon conducting a physical and neurological examination, the area of allodynia was identified at the anterior T3–4 dermatome, with no skin lesions evident on the anterior chest wall. Muscle strength in all four limbs was assessed as normal, and deep tendon reflexes did not exhibit hyperreflexia. The Babinski reflex indicated bilateral plantar flexion. The patient's gait was stable, and there were no signs of truncal ataxia or dysmetria. Proprioception testing yielded normal results. Given the clinical impression of T3–4 radiculopathy, a series of imaging studies were initiated. A plain radiograph of the spine showed signs of scoliosis, with a Cobb angle of 24.1°, which is a decrease from the 30° noted a year prior. Cervicothoracic magnetic resonance imaging (MRI) demonstrated the presence of syringomyelia spanning levels C2 to C6, as well as cerebellar tonsil herniation (Fig. 1). To further investigate the potential diagnosis of Chiari I malformation, a brain MRI was conducted, which indicated sagging of the bilateral cerebellar tonsils (Fig. 2A), the absence of aqueduct stenosis (Fig. 2B), and occult hydrocephalus characterized by an Evan's index of 0.24 (Fig. 2C) and a temporal horn width of 3.6 mm (Fig. 2D). Subsequent lumbar MRI did not reveal additional syringomyelia lesions. The radiological findings included a basal angle of 127.9°, a clivoaxial angle of 139.2°, a basilar impression of 4.5 mm, and tonsillar descent of 7.6 mm. Diagnosed with Chiari I malformation accompanied by C2 to C6 syringomyelia, the patient underwent foramen magnum decompression and duraplasty, with an uneventful surgical course. The patient was discharged on postoperative day 9, reporting improvement in allodynia. However, one week post-discharge, clear fluid was observed discharging from the surgical site, necessitating primary suture with gauze compression. Three weeks following the surgery, a brain computed tomography scan revealed a pseudomeningocele in the posterior fossa adjacent to the duraplasty site (Fig. 3A). After discussions with the patient and her family, conservative management with gauze compression was initiated. Nevertheless, the patient continued to experience persistent occipital headaches and cerebrospinal fluid leakage from the wound. A subsequent brain computed tomography scan indicated an enlargement of the pseudomeningocele with upward and epicranial extension after two weeks of conservative treatment (Fig. 3B), while no significant changes were noted in the size of the bilateral ventricular temporal horns (Fig. 4). Cervicothoracic MRI T1 revealed a tension pseudomeningocele extending into the occipital subgaleal space and partial resolution of the syringomyelia (Fig. 3C). In light of the tension pseudomeningocele complicating the foramen magnum decompression with duraplasty, a ventriculoperitoneal shunt was placed, with the programmable shunt pressure set to 100 mmH2O. One week following the shunt placement, there was a marked improvement in the pseudomeningocele and sustained resolution of the syringomyelia (Fig. 3D). In addition, improvement of headache and absence of cerebrospinal fluid leakage were noted.

## 3 Discussion

Epidural fluid accumulation is a frequently encountered complication following foramen magnum decompression with duraplasty [3]. However, the persistent accumulation of epidural fluid and the subsequent development of a pseudomeningocele represent significant challenges that require attention. The literature indicates that the progressive enlargement of the pseudomeningocele can create a mass effect, which may lead to spinal cord edema and exacerbate syringomyelia [1]. There remains no definitive consensus regarding the closure of the durotomy post-surgery for foramen magnum decompression. The durotomy is often left open to facilitate the dissipation of cerebrospinal fluid (CSF) waves or to prevent pressure on the obex by enlarging the cisterna magna, thereby creating a pseudomeningocele. Alternative techniques, such as primary dural closure, duraplasty, or the use of a patch, which are prone to failure, may counteract the benefits of decompressive surgery performed via foramen magnum decompression [6].

The precise mechanism underlying the development of pseudomeningocele as a complication of foramen magnum decompression with duraplasty in patients with Chiari I malformation remains unclear. One proposed mechanism involves the dissipation of the CSF systolic pressure wave, which shifts from the obex to the expandable cavity of the pseudomeningocele [7]. This phenomenon may explain the observed resolution of syringomyelia alongside the continued enlargement of the pseudomeningocele following foramen magnum decompression. We hypothesize that the persistent craniospinal pressure gradient across the foramen magnum may contribute to the progressive development of pseudomeningocele after decompression surgery with duraplasty. Furthermore, CSF may enter the pseudomeningocele cavity through a flap valve, which results from the dural patch sutured to the aponeurosis. In cases of deterioration, the ongoing accumulation of CSF can lead to the formation of a tension pseudomeningocele [1].

To mitigate the imbalanced pulsations of CSF at the flap valve, it may be beneficial to address the leakage of CSF through the slit of the flap valve. Prior to the complete sealing of the duraplasty site, it is essential to attenuate the pulsatile CSF pressure gradient within the pseudomeningocele cavity. Potential interventions to achieve this include external ventricular drainage, ventriculoperitoneal shunt, and endoscopic third ventriculostomy [[8], [9], [10]]. Additionally, it has been reported that 5.1 % of patients with Chiari I malformation develop hydrocephalus following foramen magnum decompression, with 17 % of these individuals experiencing CSF leakage or pseudomeningocele formation. The shunting procedure is often regarded as the preferred treatment option [4]. In our case, mild dilation of the bilateral temporal horns of the ventricles was observed prior to foramen magnum decompression, although there were no clinical signs or symptoms indicative of hydrocephalus. Nevertheless, persistent CSF leakage and the enlargement of the pseudomeningocele were noted postoperatively. Given the suspicion of hydrocephalus-related progression of the pseudomeningocele and CSF leakage, we opted to perform a programmable ventriculoperitoneal shunt. Following posterior fossa decompression, the ventricular size did not exhibit enlargement. Although the hydrocephalus was not overtly apparent prior to the decompression, it appears to have contributed to the exacerbation of the tension pseudomeningocele. Significant improvements in tension pseudomeningocele and cerebrospinal fluid leakage were observed following shunting surgery. In addition to ventriculoperitoneal shunts, various strategies exist for addressing persistent pseudomeningocele after foramen magnum decompression. A notable drawback of external ventricular drainage is the heightened risk of infection, which leads us to exclude this option [11]. Endoscopic third ventriculostomy presents an alternative; however, we refrain from selecting this procedure due to its potential complications, which include intraventricular hemorrhage, intracerebral hemorrhage, and oculomotor nerve palsy [12].

**Fig. 5:**
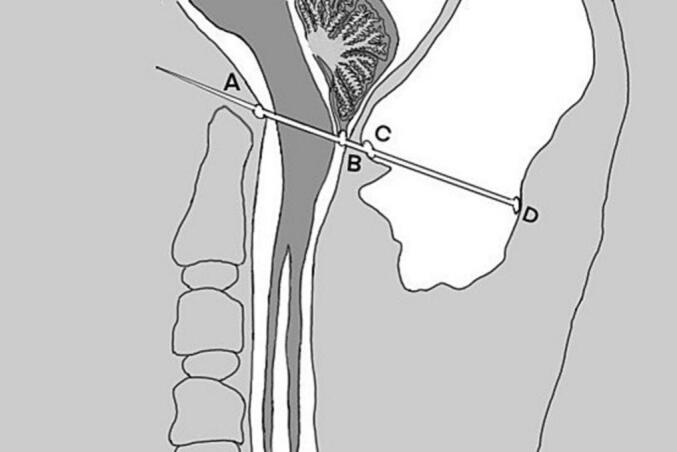
presents a schematic representation illustrating the established formula for calculating the ratio of pseudomeningocele. The components are labeled as follows: A represents the basion, B denotes the tonsil, C indicates the dura mater, and D signifies the fascia of the scalp. The line segment AB extends and intersects the dura mater and the fascia of the scalp at points C and D, respectively. The ratio of the pseudomeningocele is defined as the distance between points C and D divided by the distance between points A and D.

Despite the numerous disadvantages associated with ventriculoperitoneal shunts—such as under-drainage, over-drainage, shunt malposition, infection, subdural hematoma, and hygroma—this approach appears to be an effective solution for managing progressive pseudomeningocele following foramen magnum decompression surgery [13]. A review of the literature identified seven cases of pseudomeningocele, allowing for the evaluation of pseudomeningocele size post-posterior fossa decompression. We calculated the ratio of pseudomeningocele in the posterior fossa using a defined formula (Fig. 5). To our knowledge, we present a case with the highest ratio of pseudomeningocele in the posterior fossa (Table 1). This literature review encompasses a total of seven patients, among whom one underwent a lumbar-peritoneal shunt, one received a ventriculoperitoneal shunt, one was treated solely with aspiration, and the remaining patients underwent duraplasty. All patients exhibited improvements in clinical symptoms and neurological deficits following appropriate management.

**Table 1:** Summary of Patients Characteristics in the Literature Review.

Author	Age, years	Gender	Initial presentation	Treatment	Dural closure	PMC ratio	Presentation	Management of PMC
[14]	3	Male	.Chiari 1 .Syrinx .Kleeblattschädel deformity	Suboccipital craniectomy and C1 and C2 laminectomies	Durepair patch and running polydioxanone sutures	0.28	No clinical symptoms	Observation
[15]	22	Male	.Chiari 1 .Syrinx	Suboccipital craniectomy And C1 laminectomy	Dura was left open	0.4	PMC with cord distortion	.Draplasty with synthetic graft .Spinal cord adhesiolysis
[16]	33	Female	Chiari 1	Craniocervical decompression	–	0.55	–	–
[17]	34	Female	Chiari 1	Suboccipital craniectomy and C1 laminectomy	Duraplasty with periosteum and DuraGen XS	0.53	PMC with low-grade infection.	.Lumbar drain then lumbar peritoneal shunt . Duraplasty with muscular flap
[18]	54	Female	.Chiari 1 .Syrinx	Suboccipital craniectomy and C1 laminectomy	Duraplasty with Seamdura and running 5–0 nylon suture .polyglycolic acid sheet .fibrin glue	0.5	Headache and nausea	Aspiration
[19]	37	Male	Chiari 1	Posterior fossa decompression surgery	–	0.25	–	**–**
Lee et al.		Female	.Chiari 1 .Syrinx	Suboccipital craniectomy and C1 laminectomy	Duraplasty with galea aponeurosis + simple interrupted suture + DuraSeal	0.57	Headache + cerebrospinal leakage from the wound	Ventriculoperitoneal Shunt

Note. a: PMC as abbreviation of pseudomeningocele.

We propose that cerebrospinal fluid diversion techniques, such as pseudomeningocele-peritoneal shunt or ventriculoperitoneal shunt, may be crucial in alleviating pressure within the pseudomeningocele cavity and achieving a balance in the craniospinal pressure gradient between the wave at the obex and the pseudomeningocele cavity.

## 4 Conclusion

Tension pseudomeningocele is a possible complication that may arise after foramen magnum decompression accompanied by duraplasty in individuals diagnosed with Chiari I malformation. The mechanism contributing to this condition is likely linked to a persistent craniospinal pressure gradient between the obex and the adjacent cavity. The application of cerebrospinal fluid diversion techniques may play a crucial role in the management of tension pseudomeningocele in patients with Chiari I malformation.

## Author contribution

Yi-Chen Lee conducted the collection and analysis of the results and authored the manuscript. Yu-Feng Su provided supervision for the paper. Hui-Yuan Su undertook the review of the article and assumed responsibility for data collection and processing. All authors engaged in discussions regarding the results and contributed to the final version of the manuscript.

## Parental consent

Written informed consent was obtained from the patient's parents/legal guardian for publication and any accompanying images. A copy of the written consent is available for review by the Editor-in-Chief of this journal on request.

## Consent

The patient's parents provided written informed consent for the publication and any related images.

## Ethical approval

This study does not require ethical approval from our ethics committee.

## Guarantor

Hui-Yuan Su.

## Research registration number

Not applicable.

## SCARE guideline

The study has been conducted in accordance with the SCARE guidelines.

## Sources of funding

This research did not receive any specific grant from funding agencies in the public, commercial, or not-for-profit sectors.

## Declaration of competing interest

All the authors have no financial and non-financial conflicts of interest relevant to this article to disclose.
